# Methodological Considerations on the Randomized Trial of Self-Selected Music for Musculoskeletal Back Pain in the Emergency Department

**DOI:** 10.5811/westjem.61381

**Published:** 2026-05-19

**Authors:** Süleyman Gökhan Kara, Güneş Özlü

**Affiliations:** Eskişehir City Hospital, Department of Emergency Medicine, Eskişehir, Türkiye

Dear Editor:

We were intrigued by Goldfine et al’s “*Randomized Trial of Self-Selected Music Intervention on Pain and Anxiety in Emergency Department Patients with Musculoskeletal Back Pain*.”^1^ Such research could improve emergency department (ED) treatment for musculoskeletal back pain in a time when non-pharmacological analgesics are regarded more favorably. However, several methodological aspects of the study design and reporting may affect results interpretation and generalizability. Our findings and recommendations aim to improve this study’s scientific significance.

Although a pilot study, the 40 participant-sample size reduces statistical power and increases type II error. The investigation in one ED limits its applicability to other ED populations with different sociodemographic and cultural characteristics. Multicenter designs could solve this problem by allowing EDs with different patient demographics and operational features to compare the intervention’s effects. Despite randomization, the music group had significantly higher Pain Catastrophizing Scale (PCS) scores (28.4 [12.6] vs 19.4 [10.8], *P* =.02), showing a significant baseline difference that compromises internal validity. Even with statistical adjustments, the influence of psychosocial factors on pain perception makes it difficult to assess the intervention’s true impact. Small samples are more prone to baseline imbalances; thus, it is unclear whether the effect is attributable to music or psychological differences across groups. The PCS and anxiety-stratified randomization might improve future experiments. A significant percentage of individuals (63%) indicated prior use of music for relaxing, while 38% reported daily music listening. This familiarity with music may serve as a confounding variable affecting the reaction to the intervention; however, the distribution of these traits among the groups was not disclosed.

The authors found a significant difference in pain levels between the music and noise-cancellation groups (6.1 [0.4] vs 7.5 [0.4], *P* = .037). A 1.4-point drop on a 0–10 numeric rating scale is statistically significant but nears the smallest clinically relevant difference for musculoskeletal pain. The fact that 60% of the music group participants reported no pain change suggests that statistical significance did not match clinical relevance. Thus, future studies should include as primary objectives patient satisfaction, duration until additional analgesia is requested, and rescue analgesic use, as these may better reflect clinically significant outcomes in ED patients with back pain.

The “noise-cancellation” condition appears to be a passive control group, but in a highly stimulating environment like the ED, total silence or the reduction of ambient noise may cause sensory deprivation in some patients, which may paradoxically increase anxiety or focus on pain. In some noise-cancelling headphones, the low-frequency hum produced by active noise cancellation may cause uneasiness or restlessness. Thus, the control condition may actively alter pain and anxiety measurements. This makes music’s influence harder to pinpoint. An optimal design would include a third arm receiving standard care exclusively (without headphones and under typical ED conditions) to better distinguish between music, noise cancellation, and the natural progression of symptoms in patients with musculoskeletal back pain. Interpreting the findings requires investigating whether noise-cancelling headphones may increase pain or anxiety in the control group.

The patients’ conviction in the therapeutic value of a music intervention may have caused a placebo effect. Allowing participants to choose their own music replicates real-world practices, but the lack of genre, rhythm, tempo, or emotional valence analysis obscures the exact musical elements that may impact pain and anxiety effects. Limiting patients’ ability to skip tracks or change selections reduces the intervention’s ecological validity and may increase anxiety by making them feel they had no control. Interpreting the findings requires considering the methodological constraint of not evaluating these aspects.

The 10-minute music and noise-cancellation interventions raised questions regarding their durability in a group with an average ED stay of 6.4 hours. The measurement of outcomes immediately post-intervention makes it unclear whether any gains were sustained at clinically meaningful intervals such as 30 minutes, 60 minutes, or at discharge. This mismatch makes it difficult to distinguish between a temporary boost and a clinically significant persistent effect, and it risks disregarding subsequent “rebound” pain or anxiety escalations.

We assert that if the methodological limitations we have identified are rectified, this research avenue could produce significantly more robust evidence and provide a more substantial contribution to the management of musculoskeletal back pain in the ED.
